# Long-Term Outcomes of Patients with Coronavirus Disease 2019 at One Year after Hospital Discharge

**DOI:** 10.3390/jcm10132945

**Published:** 2021-06-30

**Authors:** Modesto M. Maestre-Muñiz, Ángel Arias, Emilia Mata-Vázquez, María Martín-Toledano, Germán López-Larramona, Ana María Ruiz-Chicote, Bárbara Nieto-Sandoval, Alfredo J. Lucendo

**Affiliations:** 1 Department of Internal Medicine, Hospital General de Tomelloso, 13700 Ciudad Real, Spain; m4modesto@gmail.com (M.M.M.-M.); emiliamata@hotmail.com (E.M.-V.); mariamtlucas@hotmail.com (M.M.-T.); germll2003@yahoo.es (G.L.-L.); aruizchicote5@yahoo.es (A.M.R.-C.); nietosandoval.barbara@gmail.com (B.N.-S.); 2 Department of Medicine and Medical Specialties, Universidad de Alcalá, 28801 Alcalá de Henares, Spain; 3 Hospital General La Mancha Centro, Research Unit, Alcázar de San Juan, 13600 Ciudad Real, Spain; angel_arias_arias81@hotmail.com; 4 Centro de Investigación Biomédica en Red de Enfermedades Hepáticas y Digestivas (CIBERehd), Spain; 5 Instituto de Investigación Sanitaria La Princesa, 28006 Madrid, Spain; 6 Department of Gastroenterology, Hospital General de Tomelloso, 13700 Ciudad Real, Spain

**Keywords:** post-acute COVID-19 syndrome, risk factors, predictors, mortality, COVID-19, Spain

## Abstract

Background: The long-term effects of COVID-19 remain largely unclear. This study aims to investigate post-acute health consequences and mortality one year after hospital discharge. Methods: All surviving adult patients who were discharged after hospital admission due to acute COVID-19 in the first wave of the pandemic underwent a comprehensive interview. Functional assessment was performed in patients aged over 65. Clinical and hospital records were reviewed and mortality causes assessed. Results: A total of 587 patients with COVID-19 were discharged from hospital, including 266 after hospital admission and 321 from the emergency room. Mortality within the following year occurred in 34/266 (12.8%) and 10/321 (3.1%), respectively, due to causes directly or possibly related to COVID-19 in 20.5% and 25% of patients. Post-COVID-19 syndrome was assessed in 543 patients at one year from discharge. Any clinical complaint was reported by 90.1% of patients who needed hospitalization and 80.4% of those discharged from the emergency room (*p* = 0.002), with breathlessness (41.6%), tiredness (35.4%), ageusia (30.2%), and anosmia (26.3%) being the most common complaints. Ongoing symptoms attributed to COVID-19 were reported by 66.8% and 49.5% of patients, respectively (*p* < 0.001). Newly developed COPD, asthma, diabetes, heart failure, and arthritis—as well as worsening of preexisting comorbidities—were found. Conclusions: One-year mortality among survivors of acute COVID-19 was 7.5%. A significant proportion of COVID-19 patients experienced ongoing symptoms at 1 year from onset of the disease.

## 1. Introduction

The coronavirus disease 2019 (COVID-19), caused by the severe acute respiratory syndrome coronavirus 2 (SARS-CoV-2) [1,2], has profoundly impacted the world, altering the functioning of populations and health systems [3,4]. Despite the high mortality seen among hospitalized patients with the acute disease, little is yet known about the medium- and long-term sequelae of severely ill COVID-19 survivors, since only a few recent studies have provided data 2–6 months post-discharge [5,6,7,8,9,10]. Patients hospitalized with COVID-19 most commonly suffer pneumonitis, with multiorgan involvement in many cases, requiring supplemental oxygen, invasive ventilation, and organ support [11]. Whilst most survivors will have a full recovery, short-term follow-up reveals that a high proportion of individuals still report fatigue or muscle weakness, dyspnea, joint pain, chest pain, and psychological symptoms, leading to impaired health-related quality of life [5,7,8,10]. Even individuals attending emergency departments but not admitted to hospital report a prolonged and debilitating set of symptoms after their acute episode, sometimes labelled as “long COVID” [9,12]—the growing recognition of which in the literature has given rise to the new concept of “post-acute COVID-19 syndrome” (PCS) [13].

Due to SARS-CoV-2 infection’s spread, and the lack of structured information about its long-term prognosis—including PCS—there is an urgent need for long-term follow-up studies on persistent symptoms, lung function, and physical and psychological problems in discharged patients [14]. Establishing the long-term persistence of the sequelae identified in the few studies that have analyzed the short-term results of COVID-19 is essential in order to allocate healthcare resources and to design specific follow-up programs to manage these patients.

Using a standardized approach, this study aims to evaluate, for the first time, the long-term consequences of the disease among survivors, after having characterized in detail a cohort of severe COVID-19 patients admitted to hospital during the first wave of the pandemic [15].

## 2. Materials and Methods

This cross-sectional study was performed at Tomelloso General Hospital—a 160-bed community medical center located in a rural area of central Spain. All adult patients with laboratory-confirmed COVID-19 who attended our hospital between 1 March and 1 June, 2020, and who were either hospitalized or discharged from the emergency room, were included. The decision to admit a patient to hospital, or to discharge them for outpatient management, was based on the provisions of the institutional care protocol developed by an expert commission at our center, which reviewed and updated the evidence on the prognosis and treatment measures for COVID-19, along with the severity of illness categories proposed by the National Institutes for Health of the USA [16].

We excluded the following patients: (1) those who died during hospital admission, (2) those for whom follow-up was not possible due to patient unwillingness to participate, and (3) those unable to be contacted. All discharged patients met uniform discharge criteria according to the institutional care protocol, which guided the therapeutic decisions of physicians from different specialties to care for patients admitted with COVID-19 under homogeneous criteria.

### 2.1. Data Source and Study Variables

Clinical data for the acute phase (from symptom onset to hospital discharge) were retrieved from electronic medical records, including demographic characteristics (e.g., age, sex, toxic habits, etc.), clinical characteristics (e.g., comorbidities, symptom onset time, and chest images), laboratory test results, and treatment administered. The disease severity was characterized by the highest seven-category scale proposed by the WHO during the hospital stay, and adapted in the institutional care protocol for COVID-19 [17]. Data were managed using a data capture file designed in Access for data collection; information was introduced prospectively in duplicate by two investigators, using electronic clinical records as source documents. Disagreements were resolved after manually consulting the source data. Data quality was reviewed periodically using front-end range and logic checks of data entry and back-end monitoring of data, using statistical software to generate data reports. Prior to final statistical analysis, the database was reviewed for inconsistencies and subsequently subjected to data cleaning. The datasets analyzed during the current study are available from the corresponding author upon reasonable request.

### 2.2. Study Procedures

All participants (or their relatives) were contacted by telephone and interviewed by a trained medical investigator from the Department of Internal Medicine. Follow-up visits were scheduled 12 ± 1 months after discharge from the hospital, as recorded in the electronic medical charts. If follow-up contact was lost, patients had three opportunities to reschedule the visit.

The follow-up consultation consisted of a comprehensive structured interview on the state of patient health, using three questionnaires: (1) on past and current symptoms, (2) an evaluation of the worsening of previous diseases or the appearance of new illnesses, and (3) an evaluation of physical or cognitive impairment. Electronic medical records from both primary care and in-hospital entries, as well as drugs prescribed throughout the previous year, were screened by the interviewer to look for potential complications from SARS-CoV-2 infection. All of the data obtained were codified on the Access database created for the study.

In the first questionnaire, participants were asked to report persistent symptoms, which consisted of those lasting beyond 20 days, and whether their onset was recent or worsened after the development of COVID-19. For the evaluation of new diseases or the worsening of previous chronic ones, patients’ anamnesis and electronic medical records were reviewed. Specifically, worsening or new onset of chronic obstructive pulmonary disease (COPD), asthma, heart failure, arthritis or spondyloarthropathy, or diabetes mellitus were considered. Widely accepted, validated questionnaires were used for disease categorization:

Patients with dyspnea were evaluated with the aid of the modified Medical Research Council (mMRC) dyspnea scale; this scale stratifies the severity of dyspnea in respiratory diseases—particularly in COPD patients—in terms of their disability, ranging from “0” (no breathlessness except with strenuous exercise) to “4” (breathlessness leaving the house or when dressing or undressing) [18]. The Global Initiative for Chronic Obstructive Lung Disease (GOLD) [19] was used to classify airflow limitation severity in patients with COPD, together with the number of exacerbations per year, the need for intensifying treatment, or the new need for oxygen use.

Asthma was assessed according to the Global Initiative for Asthma (GINA)’s treatment steps for adults [20]—a classification system for asthma severity that supports treatment decisions. GINA establishes 5 steps to be applied to patients from those who have symptoms less than twice a month and have no risk of exacerbations (Step 1) to patients with persistent exacerbations or symptoms worsening despite correct adherence to the treatment (Step 5).

Heart failure worsening was assessed via the New York Heart Association (NYHA)’s classification of dyspnea, in addition to the need for intensification of treatment. The NYHA classification—the most widely used and well-known scoring system—differentiates between 4 functional classes, from class I (no limitation for physical activity) to IV (with disability for any physical activity without discomfort) [21].

For the evaluation of arthritis, worsening in pain control was taken into account, as subjectively manifested by a patient, and by looking for worsening data in the Disease Activity Score-28 (DAS28) score [22] in the case of rheumatoid arthritis, in the Bath Ankylosing Spondylitis Disease Activity Index (BASDAI) [23] for ankylosing spondylitis, or by the need for treatment intensification in other types of arthritis. DAS28 describes the severity of rheumatoid arthritis using clinical and laboratory data; BASDAI allows the determination of the effectiveness of treatment for ankylosing spondylitis.

Diabetes mellitus (DM) assessment relied on glycated hemoglobin, the need for oral antidiabetic treatment intensification, new need or increase in the insulin dose, and the appearance of DM-related complications.

The Barthel Index (BI) [24] and the Lawton–Brody Scale [25] were used to assess the functional status of elderly patients. The BI is an ordinal scale that measures performance in activities of daily living (ADL) by scoring 10 variables describing ADL and mobility, with a higher score reflecting greater ability. The Lawton–Brody Instrumental Activities of Daily Living (IADL) Scale measures the degree of independence in instrumental activities that an elderly person maintains. Eight domains of function are measured, and patients are scored according to their highest level of functioning in the nine categories—from 0 (low function, or dependent) to 8 (high function, or independent).

Cognitive abilities were assessed by either the variation in the Red Cross Disability Scale [26] for patients who had no previous diagnosis of cognitive impairment or when this was not primary, or by the variation in Global Deterioration Scale (GDS) in cases where a previous diagnosis of Alzheimer’s disease had been provided [27]. The Red Cross Disability Scale is commonly used in geriatric centers in Spain, and qualifies stages or degrees of disability from “0” (normality) to “5” (maximum degree of deterioration). The GDS separates the course of dementia into 6 stages, ranging from 1 (no cognitive decline) 7 (very severe cognitive decline).

COVID-19-related complications assessed included the appearance of angina, myocarditis, pericarditis or acute myocardial infarction, stroke or encephalitis, pleuritis or pleural effusion, thromboembolic disease, antiphospholipid syndrome, pemphigus or psoriasis, and acute renal failure [28,29].

### 2.3. Study Procedures

Means ± SDs or medians ± interquartile ranges (IQRs) were used for continuous variables, and percentages for categorical variables. Comparisons between patients admitted to hospital and patients discharged from the emergency room were performed using Student’s *t*-test or the Mann–Whitney U test for continuous variables, and the χ^2^ test or Fisher’s exact test for categorical variables. For comparison between the initial discharge and the 1 year follow-up, a non-parametric paired Wilcoxon signed-rank test was used. All analyses were carried out using SPPS v.18.0. A *p*-value < 0.05 was considered to be significant.

### 2.4. Ethics

The study was conducted in accordance with the guidelines of the Declaration of Helsinki; the database supporting data collection was approved by the Ethics Committee of La Mancha-Centro General Hospital at Alcázar de San Juan (protocol code 142-C) on 24 May 2020.

## 3. Results

### 3.1. Demographic and Clinical Characteristics of Hospitalized and Discharged Patients

**Figure 1:**
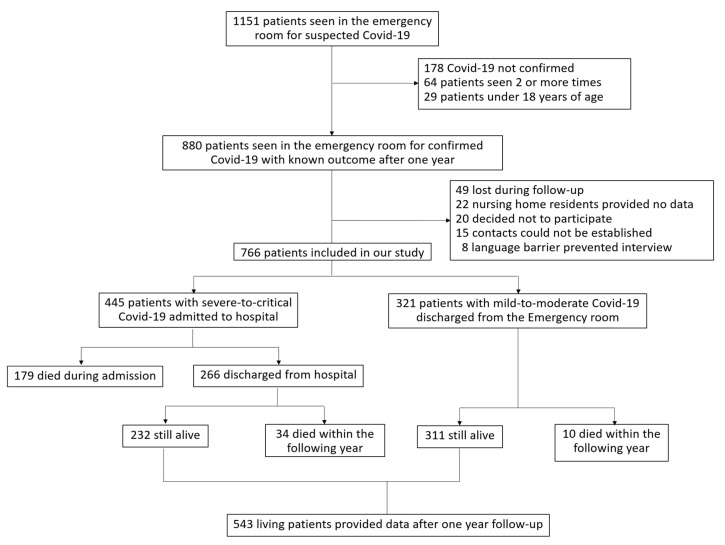
Flow chart of patients who attended Tomelloso General Hospital during the first wave of the COVID-19 pandemic, and who were admitted to hospital or discharged from the emergency room. After one year, patients were evaluated for the long-term consequences of the disease.

A total of 766 patients with COVID-19, who attended Tomelloso General Hospital during the study period (1 March to 1 June 2020), had information on their clinical outcomes available after one year. Follow-up was completed from 15 March to 10 May 2021 in 543 patients. Overall, 321 patients with mild-to-moderate COVID-19 were discharged directly from the emergency room, and 445 severe-to-critical patients were admitted to hospital. Among them, 432 patients (97.1%) presented with pneumonia and 417 (93.7%) required supplemental O_2_. One-hundred and seventy-nine admitted patients (40.2%) died, and 266 were discharged after a median ± IQR stay of 8 days ± 10 days (rank 1–115 days). Figure 1 describes the flow of study patients throughout the year after acute COVID-19.

Demographic and clinical characteristics of all patients who attended our site, and for whom we have data on their clinical outcomes or a complete follow-up, are summarized in Table 1. The patients admitted to hospital were significantly older that those discharged from the emergency room (the mean ± SD age being 71.5 ± 14.3 and 56.2 ± 17.8 years, respectively, *p* < 0.001), and presented concomitant diseases with significantly higher frequency; hypertension was the most common (67.4%), followed by diabetes (33.7%), dyslipidemia (30.6%), and obesity (21.1%). Overall, 89.9% of hospitalized patients presented any comorbidity, compared to 61.1% of patients who were discharged from the emergency room. Nursing home residents were preferably distributed among patients admitted to hospital (14.4%) compared to those discharged from the emergency room (3.4%) (*p* < 0.001).

### 3.2. One-Year Mortality Rates after COVID-19 Discharge and Causes of Death

Of the 266 patients who were admitted to hospital and later discharged, 34 (12.8%) died during the following year. In contrast, only 10 (3.1%) of the 321 COVID-19 patients who were discharged from the emergency room died before follow-up. Median ± IQR time from acute COVID-19 discharge to death was 68 ± 135 days (rank 4–264). Of the 44 patients who died, 9 (20.5%) died from respiratory failure clearly related to COVID-19 pneumonia, and 20 (45.4%) died due to non-COVID-19-related causes—mainly aspiration pneumonia, bacterial pneumonia, and complications from previous illness or tumor progression. Additionally, 11 patients (25%) died from complications possibly related to COVID-19, including acute myocardial infarction, non-ischemic heart failure, sudden death with a suspected relationship to malignant arrhythmia, pulmonary thromboembolism, and hemorrhagic stroke. Finally, the cause of death in the last 4 patients could not be determined. Supplementary Table S1 summarizes causes of death in patients within the following year after hospital discharge, and their possible relationships with COVID-19.

### 3.3. Recovered and Ongoing Symptoms after COVID-19

Any clinical complaint within the year after acute COVID-19 was reported by 84.5% (459/543) of patients, the higher proportion being in those who needed hospital admission (90.1%) compared to those discharged from the emergency room (80.4%; *p* = 0.002).

At 12 months from discharge, none of the patients had residual fever or any signs or symptoms of acute illness; however, 56.9% (309/543) of patients still presented ongoing symptoms attributed to PCS; this proportion was higher in patients who needed hospital admission (66.8%) than in those who were discharged from the emergency room with acute COVID-19 (49.5%; *p* < 0.001).

COVID-19 patients who did not require admission more frequently presented loss of smell (anosmia) (29.6% vs. 22%; *p* = 0.047), loss of taste (ageusia) (35.7% vs. 22.8%; *p* = 0.001), and a sore throat (7.7% vs. 3%; *p* = 0.020). Headache, low-grade fever, chest pain, and gastrointestinal symptoms (diarrhea, vomiting, or abdominal pain) tended to be non-significantly more frequent among patients discharged without admission. All of the other complaints were more frequent among patients who required admission. Patients with anosmia and ageusia usually recovered after a median time of 31 days, but some degree of ageusia and anosmia were still present in 23.8% and 27.3% of patients overall, respectively, with no significant differences between admitted patients and outpatients.

As for headaches, 50.5% of the patients with this symptom reported suffering from reoccurring episodes more frequently than before SARS-CoV-2 infection, with the proportion being higher among patients who were hospitalized (68.6% vs. 40.3%; *p* = 0.008). The duration of gastrointestinal symptoms showed no differences according to patients’ type, and persisted in 9.5% of patients one year after infection. Persistent sore throat was more common among patients who were admitted to hospital (71.4% vs. 29.2%; *p* = 0.043).

Overall, 226 of the 543 patients evaluated (46.5%) suffered breathlessness at some point after discharge. This was more common in patients admitted to hospital (53.9%) than in those who were discharged from the emergency room (32.5%; *p* < 0.001). At the point of follow-up, some degree of breathlessness was still present in 105 of these 226 patients (54.7%), with no differences between patient type (admitted to hospital or outpatient). Inpatients recovered from breathlessness after a median ± IQR of 36.5 ± 66.3 days, with no differences between patient type.

Overall, 35.4% of patients reported tiredness, and 18.6% reported muscular weakness, with both symptoms being present in similar proportions in patients admitted to hospital and outpatients, and persisting one year post-COVID-19 in 54.7% and 39.6%, respectively. Recovery time was longer for patients admitted to hospital than for patients discharged from the emergency room (39 ± 71 vs. 18.5 ± 51 days; *p* = 0.018). A total of 16% of patients reported having suffered from myalgia at some point during this period (19.8% vs. 13.2%, respectively, *p* = 0.037), which still persisted at one year in 70.5% of cases, with no differences overall in patient type.

Regarding neurological symptoms: 20.8% and 19.3% of patients overall reported memory lapses and insomnia throughout the previous year. These persisted in most patients one year after acute COVID-19, and were more common among hospitalized patients (26.7% vs. 16.4%, *p* = 0.003).

**Figure 2:**
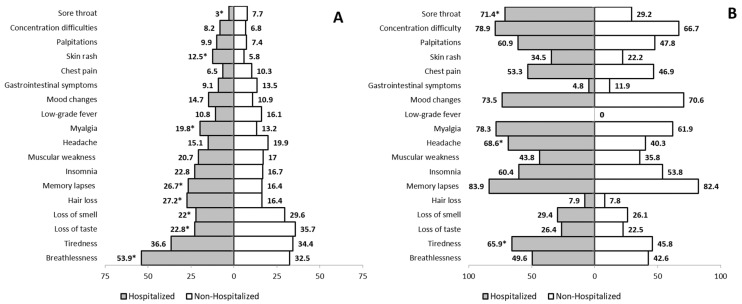
Percentages of patients presenting with specific coronavirus disease 2019 (COVID-19)-related symptoms at any point during the year after the acute phase of the disease (**A**); and continuation of the reported symptoms one year after acute COVID-19 in variable proportions (**B**). * denotes statistically significant differences (*p* < 0.05).

Other symptoms reported in a lesser proportion of patients are summarized in Figure 2. Detailed information on the main clinical symptoms reported by patients recovered from acute COVID-19 at one-year follow-up, as well as recovery time (in days with median (IQR)) and the proportion of patients still with ongoing symptoms at the moment of assessment, is provided in Supplementary Table S2.

### 3.4. Complications after Acute COVID-19

In the year following COVID-19 infection, 13 patients overall presented with deep venous thrombosis, and 9 with pulmonary thromboembolisms (with two patients having both diseases); no differences between hospital-admitted and emergency-room-discharged patients were found in the occurrence of this complication.

Among surviving patients, 8 (1.5%) presented with acute myocardial infarction, and 7 (1.3%) with stroke, with the distribution being similar in both patient groups. In each group, 1 patient developed myocarditis (0.3%), and 4 more (0.7%) developed pleural effusion unrelated to pneumonia—3 of whom were among post-COVID-19 patients admitted to hospital.

A total of 13 patients (2.4%) had newly developed arthritis, equally distributed among both patient type groups. Transient acute kidney injury appeared in 4 patients who had been admitted to hospital due to severe COVID-19, and 1 more patient within this group presented acute encephalitis. Table 2 provides additional details on post-acute-COVID-19 complications.

### 3.5. Worsening of Concomitant Diseases during the Year after COVID-19

A total of 10 patients (1.8%) were newly diagnosed with COPD after recovering from acute COVID-19 (who were distributed between GOLD grades 1 and 2 at 50%). In 11 patients who already had COPD prior to COVID-19, at-home oxygen therapy was newly required (39.3%) after assessment of blood gases, and 7 patients required treatment augmentation. A chest radiograph was performed in 89 of the 105 patients with persistent dyspnea/breathlessness after recovering from acute COVID-19; changes suggestive for diffuse or localized interstitial fibrosis were found in 8 (9%) and 18 patients (20.2%), respectively; of these patients, 18 underwent high-resolution computed tomography (HRCT), which confirmed diffuse or irregularly distributed fibrotic lung changes in 6 (33.3%) and 5 patients (27.8%), respectively.

Asthma was developed by 2 patients (0.4%) during the year after acute COVID-19 recovery. Asthma therapy was augmented in 8 additional former asthmatic patients (20.5%).

Diabetes onset was diagnosed in the post-COVID-19 recovery year in 7 patients (1.3%). In addition, intensification of oral antidiabetic drugs or insulin was respectively required in 10 and 5 previously known diabetic patients. A further 2 diabetic patients were diagnosed with peripheral neuropathy, and 3 more with retinopathy, after recovery from acute COVID-19.

Heart failure onset was reported in 11 of the patients (2.0%) who recovered from COVID-19. Another 7 patients with previously known chronic heart failure required treatment augmentation after suffering from COVID-19.

A previously inadvertent onset of rheumatic disease was found in 4 patients; 3 patients with arthritis/spondylitis reported worsening control, and another needed treatment intensification. Additional details are provided in Table 3.

Among the 69 patients who had been previously diagnosed with cognitive impairment (12 with Alzheimer’s disease (AD) and 57 with a different origin), one-year assessment was available for 35. Among AD patients, 5 were clinically impaired during the year after acute COVID-19 (42%), and 7 maintained their previous cognitive status. Among the 23 patients with dementia other than AD who could be evaluated for this study, cognitive impairment was found in 14 (61%), with the remaining 9 maintaining cognitive functioning.

Functional status was assessed in the 264 patients aged over 65; a functional decline according to the BI was noted in 22 of them (8.3%). The Red Cross Disability Scale detected cognitive impairment in 14 of these patients. A loss in patient autonomy was detected in 21 patients according to the Lawton–Brody IADL Scale, which reduced from a median ± IQR of 3.5 ± 4 to 1.5 ± 5 during the post-COVID-19 year (*p* = 0.017). Finally, a non-significant decline in median ± IQR GDS scores from 4.5 ± 3 to 5 ± 2 was noted in the 12 patients who had a diagnosis of Alzheimer’s disease. No additional patient was diagnosed with dementia during the study period.

## 4. Discussion

To our knowledge, this cohort study, which adds to the limited literature available on the medium-term sequelae of COVID-19, has the longest follow-up duration assessing the health consequences of adult patients discharged from hospital recovering from COVID-19.

Our research was carried out on a series of seriously ill patients, with a hospitalization rate of 58.1%, and with a prevalence of pneumonia of 74.4%. In these conditions, overall mortality within the first year after SARS-CoV-2 infection reached 29.1% (223/766). The recommendations of the Spanish authorities during the first wave of the pandemic for patients to stay at home, even if they had symptoms that were currently considered serious and indicative of a poor prognosis, could certainly be a contributing factor to these results. In line with previous publications [30,31,32], more comorbidities were expected in hospital-admitted patients compared to emergency-room-discharged patients. Virtually all comorbidities were more frequent in the former, and their prevalence rates in our series were higher than those reported in other studies [10], as well as higher than those expected for the general population in Spain, according to figures provided by the National Institute of Statistics. Thus, 67.4% of hospitalized patients presented with hypertension (which affects up to 19.8% of people in Spain), 24.7% suffered from diabetes mellitus, 24% from hyperlipidemia, and 18.1% were overweight or obese. These figures are 7.8%, 17.9%, and 17.1%, respectively, in the overall Spanish population [33,34]. The high prevalence of hypothyroidism and depressive syndrome found in our population requires a special mention; these are not described as common comorbidities in most COVID-19 reports; however, Brix et al. [35] did report impaired prognosis of COVID-19 among patients with hypothyroidism.

Remarkably, mortality after hospital discharge and during the 1-year study period was as high as 7.5%, which contrasts with the 1.5% mortality rate reported at month 6 among survivors of COVID-19 in Wuhan, China [10], which was mainly caused by exacerbation of underlying pulmonary, heart, and kidney diseases. In half of the patients, causes of death could likely or possibly be related to complications from SARS-CoV-2 infection. A hypercoagulability mechanism could explain deaths in all of these patients [36].

Persistent symptoms related to COVID-19 infection (the so-called PCS) have recently become a popular research topic. PCS has been defined by the persistence of at least one clinically relevant symptom, spirometry disturbance, or significant radiological alteration in post-COVID-19 patients [37], and it has been proposed as being related to residual inflammation, organ damage, non-specific effects from hospitalization or prolonged ventilation, social isolation, or disease impact on pre-existing health conditions. According to the proposed definition of PCS, most of our patients (84.5%) fulfilled the diagnostic criteria at any given point, and 56.9% still suffered from PCS one year after acute infection. Symptoms involved in PCS show wide heterogeneity across the literature in terms of type and frequency, and also vary according to the time they are assessed. Thus, Tendforde et al. evaluated outpatients after 2–3 weeks from the COVID-19 diagnostic test, and found that 94% of them still had symptoms, with fatigue (71%), a cough (61%), and headache (61%) being the most common [38]. Halpin et al. via a structured telephone interview carried out 4–8 weeks after hospital discharge, described fatigue in up to 72%, breathlessness in up to 65%, and psychological distress in up to 46.9% of patients who recovered from acute COVID-19 [39]. Moreno-Pérez et al. evaluated the presence of PCS 10–14 weeks after outpatient COVID-19 recovery or discharge from hospital [35]; around 50% of patients presented persistent symptoms, the most frequent being fatigue (35%), dyspnea (34.4%), headache (16.8%), anosmia/dysgeusia (21.4%), and mnesic complaints (15.2%). In the longest follow-up assessment available until now [10], Huang et al. described 76% of patients overall reporting at least one symptom at 6 months after recovery from acute disease, which included fatigue or muscle weakness (63%), sleep difficulties (26%), mood disorders (anxiety 23%, depression 22%), hair loss (11%), smell disorder (11%), joint paint (9%), and headache (2%). These figures mirror our findings, in that four out of five patients had persistent complaints within the year after recovering from acute COVID-19, and more than half reported already having ongoing symptoms at the time of assessment. The most common PCS complaints were breathlessness (41.6%), tiredness (35.4%) or muscle weakness (18.6%), loss of taste and smell (25%), hair loss (21%), and memory lapses (20.8%). Multiorgan impairment after acute COVID-19 has been involved in the long-term persistence of symptoms [40], and low-level inflammation of the heart, kidneys, liver, pancreas, and lungs has been shown by using magnetic resonance imaging, leading to reductions in organ function and providing objective evidence that PCS represents a multisystem syndrome rather than a single condition.

Hospital readmission due to potential complications from COVID-19 during the study period, especially those related to thrombotic events, occurred in 3.9% of our patients, consistent with data reported by Salisbury et al. [41]: A 90-day follow-up of all patients admitted to hospital confirmed a 5.9% rate of venous thromboembolism events, despite patients being under standard thromboprophylaxis. Once again, heterogeneous data have been provided in this regard, with additional reports describing only 3 out of 2469 patients suffering a stroke, and only 1 suffering a pulmonary embolism, in a 6-month follow-up period [10]. As for acute myocardial infarction or ischemic stroke, our incidence was found to be 1.5% and 1.3%, respectively, consistent with the findings of Modin et al. [42], who described a 5–10-fold increase in the incidence of both diagnoses during the pandemic compared to a control period. Autopsies of COVID-19-deceased patients revealed myocarditis and myocardial infarctions to be present in 7.2% and 4.7%, respectively [43]. Two patients in our series presented symptomatic myocarditis that required hospital admission.

Complications with an underlying inflammatory basis were also found, including pleural effusion (0.7%) and inflammatory arthritis (2.4%); the latter appeared de novo in patients with no previous history, and has been described as affecting 1.9% of patients hospitalized due to acute COVID-19 [44], all due to microcrystals. SARS-CoV-2 as an infectious trigger of reactive arthritis has also been proposed [45], helping to explain the impaired control of previous arthroplasties in 11% of our patients. In fact, a significant increase in several serum biomarkers involved in inflammation and stress responses has been described to long persist in patients with COVID-19, even in milder forms [46]. Pleural effusion after COVID-19 unrelated to venous thromboembolism, heart failure, or pneumonia is suspected to be directly related to SARS-CoV-2 infection, as supported by the detection of the virus through RT-PCR in the pleural effusions of patients [47].

Interestingly, no differences in the frequency of complications were found in relation to the degree of severity of the disease, except for acute renal failure, which exclusively manifested in four subjects who required hospital admission (0.7%). Acute kidney insufficiency represents a common and serious complication of acute COVID-19 and an independent risk factor for mortality [48]. Its evolution over time has not been well established, but probably leads to a persistent reduction in glomerular filtration rate after week 24 of discharge among patients who developed in-hospital acute kidney insufficiency [49].

Our study also evaluated the onset or worsening of previous diseases among patients who had recovered from acute COVID-19. To begin with, 2% of patients developed de novo arterial hypertension during the study year, which can be explained by the interference SARS-CoV-2 has over the rennin angiotensin system (RAS), with angiotensin-converting enzyme 2 (ACE2) being the major counter-regulatory mechanism for the main axis of the RAS, and critical for the control of blood pressure and electrolyte balance. SARS-CoV-2 binds with ACE2 and enhances its degradation, thus decreasing the counteraction of ACE2 on the RAS, with an increased reabsorption of sodium and water, leading to increased blood pressure and excretion of potassium [50,51]. In addition, 2.7% of our study population developed right heart failure, without having any clear previous risk factors, and not linked to the development of hypertension or left heart failure. This could be a consequence of either impaired right ventricular longitudinal strain (RVLS)—as proposed by Nuzzi et al. after finding that 42% of patients who had recovered from COVID-19 without pulmonary hypertension had RVLS [52]—or the development of pulmonary hypertension. In fact, pulmonary hypertension was found in 12% of COVID-19 patients admitted to the ICU [53]. As the lungs are a primary target for COVID-19, with varying degrees of impact, newly developed COPD after infection was documented in 10 (1.8%) patients, and 17 more (60.7%) required treatment intensification. The physiological mechanisms leading to persistent dyspnea in non-critically ill COVID-19 survivors have been recently analyzed [54], and a significant decrease in forced vital capacity (FVC), forced expiratory volume in the first second (FEV1), and diffusing capacity of the lung of carbon monoxide (DLCO) after 6 min’ walking distance was noted among patients with persistent dyspnea when examined 30–60 days after the onset of COVID-19 symptoms. The respiratory outcomes in SARS and MERS—the two previous viral infection outbreaks similar to COVID-19—were systematically reviewed: after 12 months, 32.4% of sufferers who recovered presented impaired FEV1, and 29.8% had a decrease in FVC [55], which parallels the 22.6% functional impairment documented in COPD patients. In addition, several studies have shown that the occurrence of COPD exacerbations increases the risk of myocardial infarction and stroke, as well as all-cause mortality, in the post-exacerbation period—especially in the case of associated pneumonia. Consequently, a management plan for survivors of COVID-19 has been recognized as being urgently needed [56]. Findings of pulmonary fibrosis were confirmed via HRCT in a proportion of our patients with persistent dyspnea after recovery from acute COVID-19. Residual pulmonary fibrosis in HRCT has been found in up to half of COVID-19 survivors after discharge [57], its severity mainly depending on the patient’s age, comorbidities, and length of ICU stay [58]. Finally, diabetes onset or a worsening in glycemic control was documented in 1.3% and 10.1% of patients, respectively. The effect of SARS-CoV-2 in accelerating type 2 diabetes by binding the ACE2 receptor on the islet endothelial/pericyte microvasculature and the pancreatic islet cells has been described [59], with an ensuing viral–virion binding, excessive oxidative stress, and local cytokine storm that lead to B-cell dysfunction and apoptosis.

A significant loss of autonomy in daily life and instrumental activities in people aged over 65 was documented. A disruption in the blood–brain barrier caused by inflammation, and leading to increased permeability for cytokines to enter the central nervous system, has been proposed in COVID-19 survivors [60]. Neuroinflammation from microglial activation and oxidative stress might contribute to delirium in the short term and severe cognitive and functional decline in the long term. A recent investigation indicates the magnitude of the decline in functional status and health-related quality of life in people aged 60 and older, 6 months after hospital admission [61]: by using the EuroQol 5-dimensional 5-level (EQ-5D-5L) questionnaire, the authors found impaired ability to perform day-to-day activities (35%), reduced mobility (33%), increased pain or discomfort (33%), and negative changes in cognitive function (43%) compared to before the COVID-19 infection. The use of different questionnaires prevents direct comparisons with the results of our study.

Cognitive impairment in patients with previous Alzheimer’s disease or other previously acquired cognitive impairment or dementia was documented in half of the patients we assessed. The literature has described similar proportions of cognitive decline in COVID-19 survivors with dementia [62]; these data can be explained not only by a direct involvement with the central nervous system, but also by the social consequences of the pandemic—such as a greater level of stress, and the loneliness and isolation experienced by people with dementia during this period [63].

Some weaknesses of our study should be acknowledged: Firstly, the data used are from a single center, with a rural reference population and with a higher average age than in urban settings. Secondly, clinical status was assessed by means of telephone interviews, and despite their structured fashion and being supported by clinical records, a component of subjectivity cannot be excluded. Finally, a quality-of-life questionnaire was not applied, thus reducing the comparability of our results.

However, the strength of our study is in exhaustively including all patients who attended our hospital during the first wave of the pandemic, with follow-up data being obtained from the vast majority. A comprehensive assessment was made by internal medicine specialists using validated scales on subjective clinical situations, the new appearance or worsening of previous diseases, cause of death and, in older patients, their functional and cognitive status.

## 5. Conclusions

In conclusion, with the longest follow-up reported to date, this study provides evidence that COVID-19 is not just an acute infection, but a syndrome that affects a significant proportion of survivors long term, even when the initial infection was mild. In order to mitigate the effects of the pandemic, establishing programs to provide long-term follow-up of patients, as well as education to recognize symptoms indicating post-acute-COVID-19-related complications, is necessary.

## Figures and Tables

**Table 1:** Demographic and baseline comorbidities of COVID-19 patients attending our hospital during the first wave of the pandemic (1 March to 1 June 2020), and for whom we had information on clinical outcomes after a year. Characteristics of patients who were discharged from the emergency room and those who were admitted to hospital are compared.

		Total (*n* = 766)	Discharged from the Emergency Room (*n* = 321)	Admitted to Hospital (*n* = 445)	*p*-Value
Mean age at admission, years (SD; rank)		65.1 (17.5; 18–98)	56.2 (17.8; 18–96)	71.5 (14.3; 20–98)	<0.001
Age group	Over 65 years old, *n* (%)	418 (54.6%)	104 (32.4%)	314 (70.6%)	<0.001
	Younger than 65 years old, *n* (%)	348 (45.4%)	217 (67.6%)	131 (29.4%)	
Sex	Male, *n* (%)	388 (50.7)	177 (55.1)	201 (45.2)	0.006
	Female, *n* (%)	378 (49.3)	144 (44.9)	244 (54.8)	
Nursing home residents, *n* (%)		75 (9.8)	11 (3.4)	64 (14.4)	<0.001
Previous comorbidities	Arterial hypertension, *n* (%)	401 (52.3)	101 (31.5)	300 (67.4)	<0.001
	Diabetes, *n* (%)	189 (24.7)	39 (12.1)	150 (33.7)	<0.001
	Dyslipidemia, *n* (%)	184 (24)	48 (15)	136 (30.6)	<0.001
	Obesity, *n* (%)	139 (18.1)	45 (14)	94 (21.1)	0.012
	Chronic kidney disease, *n* (%)	70 (9.1)	9 (2.8)	61 (13.7)	<0.001
	Cognitive impairment, *n* (%)	69 (9)	15 (4.7)	54 (12.1)	<0.001
	Ischemic heart disease, *n* (%)	66 (8.6%)	7 (2.2%)	59 (13.3)	<0.001
	Oncological disease, *n* (%)	66 (8.6%)	18 (5.6%)	48 (10.8%)	0.012
	Sleep apnea/hypopnea syndrome, *n* (%)	62 (8.1%)	17 (5.3%)	45 (10.1%)	0.016
	Hypothyroidism, *n* (%)	61 (8%)	22 (6.9%)	39 (8.8%)	0.335
	Cerebrovascular diseases, *n* (%)	56 (7.3%)	9 (2.8%)	47 (10.6%)	<0.001
	Depressive syndrome, *n* (%)	54 (7%)	18 (5.6%)	36 (8.1%)	0.185
	Chronic obstructive pulmonary disease, *n* (%)	53 (6.9%)	11 (3.4%)	42 (9.4%)	0.001
	Bronchial asthma, *n* (%)	51 (6.7%)	25 (7.8%)	26 (5.8%)	0.287
	Anemia, *n* (%)	37 (4.8%)	8 (2.5%)	29 (6.5%)	0.010
	Chronic liver disease, *n* (%)	34 (4.4%)	8 (2.5%)	26 (5.8%)	0.026
	Congestive heart failure, *n* (%)	34 (4.4%)	3 (0.9%)	31 (7%)	<0.001
	Connective tissue disorder, *n* (%)	26 (3.4%)	12 (3.7%)	14 (3.1%)	0.655
	Blood dyscrasias, *n* (%)	26 (3.4%)	5 (1.6%)	21 (4.7%)	0.017
	Peripheral vascular disease, *n* (%)	18 (2.3%)	3 (0.9%)	15 (3.4%)	0.028
	Hyperuricemia, *n* (%)	26 (3.4%)	8 (2.5%)	18 (4%)	0.242
	Any previous comorbidity, *n* (%)	596 (77.8%)	196 (61.1%)	400 (89.9%)	<0.001

**Table 2:** Complications reported within the first year after recovering from acute COVID-19 in patients attending Tomelloso General Hospital during the first wave of the pandemic.

Complication	Total (*n* = 543)	Discharged from the Emergency Room (*n* = 311)	Admitted to Hospital (*n* = 232)	*p*-Value
Deep venous thrombosis	13 (2.4%)	5 (1.6%)	8 (3.4%)	0.165
Pulmonary thromboembolism	9 (1.7%)	4 (1.3%)	5 (2.2%)	0.507
High blood pressure onset	12 (2.2%)	8 (2.6%)	4 (1.7%)	0.506
Diabetes debut	6 (1.1%)	3 (1%)	3 (1.3%)	0.704
Pleural effusion	4 (0.7%)	1 (0.3%)	3 (1.3%)	0.318
Acute myocardial infarction	8 (1.5%)	6 (1.9%)	2 (0.9%)	0.476
Myocarditis or pericarditis	2 (0.4%)	1 (0.3%)	1 (0.4%)	>0.999
Stroke	7 (1.3%)	4 (1.3%)	3 (1.3%)	>0.999
Encephalitis	1 (0.2%)	0	1 (0.4%)	0.427
Renal failure	4 (0.7%)	0	4 (1.7%)	0.033
Arthritis	13 (2.4%)	7 (2.3%)	6 (2.6%)	0.800

**Table 3:** Clinical worsening and new onset of concomitant diseases reported by patients after one year of recovery from acute COVID-19.

Diseases	Global (*n* = 543)
Chronic obstructive pulmonary disease (COPD)	*n* (%)
Newly diagnosed from COPD	10 (1.8%)
Patients with COPD patients alive after one year/at baseline	28/53 (53%)
New need of oxygen	11/28 (39.3%)
Treatment intensification	7/28 (25%)
New need of oxygen or treatment intensification	17/28 (60.7%)
Bronchial asthma	
New appearance of asthma	2 (0.4%)
Patients with asthma alive after one year/at baseline	39/51 (76.5%)
Treatment intensification	8/39 (20.5%)
Diabetes	
New debut of diabetes	7 (1.3%)
Impaired glycemic control	11 (2%)
Patients with diabetes alive after one year/at baseline	109/189 (57.7%)
Impaired glycemic control	11/109 (10.1%)
Intensification of oral therapy	10/109 (9.2%)
New prescription of insulin	5/109 (4.6%)
Development of peripheral neuropathy	2/109 (2.7%)
Development of retinopathy	3/109 (1.8%)
Heart failure	
New development of heart failure	11 (2%)
Treatment intensification	7
Arthritis/Spondyloarthropathy	
New development of Arthritis/Spondyloarthropathy	4 (0.7%)
Worsening pain control	3
Treatment intensification	1

## Data Availability

Data that support this request may be provided after a reasoned request.

## Supplementary Materials

The following are available online at https://www.mdpi.com/article/10.3390/jcm10132945/s1. Table S1: Patients who died within the year after being discharged from hospital after acute COVID-19.
