# Supplementary material for: Long-Term Outcomes of Patients with Coronavirus Disease 2019 at One Year after Hospital Discharge
Source: J Clin Med. 2021 Jun 30;10(13):2945. doi: 10.3390/jcm10132945 (PMC8269002; doi:10.3390/jcm10132945)
Supplement: Supplementary file 1 [file jcm-10-02945-s001.zip › Table S1.pdf]

**Table S1.** Patients who died within the year after being discharged from hospital after acute Covid-19

| Related to Covid-19 | Sex | Age | Management during acute Covid-19 | Time from discharge to death (days) | Cause of death |
|---|---|---|---|---|---|
| **Likely** (n=9; 20.4%) | F | 92 | In-hospital | 32 | Progressive respiratory failure after Covid-19 pneumonia |
| | F | 71 | At home | 26 | Progressive respiratory failure after Covid-19 pneumonia |
| | M | 86 | In-hospital | 23 | Progressive respiratory failure after Covid-19 pneumonia |
| | M | 87 | In-hospital | 22 | Progressive respiratory failure after Covid-19 pneumonia |
| | M | 83 | In-hospital | 13 | Progressive respiratory failure after Covid-19 pneumonia |
| | M | 86 | In-hospital | 11 | Progressive respiratory failure after Covid-19 pneumonia |
| | F | 83 | In-hospital | 14 | Progressive respiratory failure after Covid-19 pneumonia |
| | F | 86 | In-hospital | 4 | Progressive respiratory failure after Covid-19 pneumonia |
| | F | 82 | At home | 13 | Progressive respiratory failure after Covid-19 pneumonia |
| **Unlikely** (n=20; 45.4%) | M | 90 | In-hospital | 72 | Aspiration pneumonia |
| | F | 85 | In-hospital | 93 | Aspiration pneumonia |
| | M | 67 | In-hospital | 76 | Bacterial infection (pneumonia) and sepsis |
| | F | 96 | In-hospital | 217 | Anorexia, dehydration, uremic encephalopathy |
| | F | 68 | At home | 230 | Progression of blood dyscrasia and complications |
| | M | 59 | In-hospital | 178 | Bacterial infections (pneumonia) |
| | F | 92 | In-hospital | 165 | Natural cause |
| | M | 82 | In-hospital | 152 | Aspiration pneumonia |
| | F | 75 | In-hospital | 135 | Neoplasm progression and its complications |
| | M | 96 | At home | 162 | Acute chronic heart failure. Pneumonia |
| | M | 88 | At home | 117 | Bacterial infections (urinary origin sepsis) |
| | F | 82 | In-hospital | 53 | Fortuitous fall and related complications |
| | M | 85 | At home | 92 | Aspiration pneumonia. |
| | M | 89 | In-hospital | 44 | Acute chronic respiratory failure. Respiratory acidosis |
| | M | 90 | In-hospital | 72 | Aspiration of epistaxis, atrial fibrillation |
| | M | 69 | In-hospital | 33 | Neoplasm progression and its complications |
| | M | 85 | In-hospital | 44 | Congestive heart failure Previous heart disease |
| | M | 80 | In-hospital | 22 | Surgery-derived complications |
| | M | 79 | In-hospital | 20 | Bacterial infections (pneumonia) |
| | F | 65 | In-hospital | 19 | Acute chronic respiratory failure. Respiratory acidosis |
| **Possibly** (n=11; 25%) | F | 87 | At home | 79 | Congestive heart failure (*de novo*) |
| | F | 91 | In-hospital | 192 | Acute left heart failure, previously unknown |
| | M | 66 | At home | 195 | Hemorrhagic stroke |
| | M | 82 | At home | 26 4 | Acute myocardial infarction |
| | F | 84 | In-hospital | 188 | Acute myocardial infarction |
| | M | 65 | In-hospital | 159 | Acute myocardial infarction |
| | F | 97 | In-hospital | 76 | Arrhythmia and syncope |
| | F | 89 | In-hospital | 40 | Acute left heart failure, previously unknown |
| | M | 93 | In-hospital | 28 | Sudden death |
| | M | 87 | In-hospital | 17 | Pulmonar thromboembolism |
| | F | 91 | In-hospital | 17 | Acute left heart failure not known |
| **Unknown** (n=4; 9.1%) | M | 60 | In-hospital | 68 | Unknown |
| | M | 85 | In-hospital | 44 | Unknown |
| | M | 90 | At home | 177 | Unknown |
| | M | 60 | In-hospital | 68 | Unknown |
