# Supplementary material for: Long-Term Outcomes of Patients with Coronavirus Disease 2019 at One Year after Hospital Discharge
Source: J Clin Med. 2021 Jun 30;10(13):2945. doi: 10.3390/jcm10132945 (PMC8269002; doi:10.3390/jcm10132945)
Supplement: Supplementary file 1 [file jcm-10-02945-s001.zip › Table S2.pdf]

Table S2. Main clinical symptoms reported by patients recovered from acute Covid-19 at one-year follow-up.

Symptom	Total (n = 543)	Discharged from the Emergency Room (n = 311)	Admitted to Hospital (n = 232)	*p* Value
Breathlessness, n (%)	226 (41.6%)	101 (32.5%)	125 (53.9%)	**<0.001**
Recovery time [median (IQR)], days	36.5 (66.3)	31 (72)	42.5 (62)	0.211
Still ongoing, n (%)	105 (46.5%)	43 (42.6%)	62 (49.6%)	0.292
Tiredness, n (%)	192 (35.4%)	107 (34.4%)	85 (36.6%)	0.590
Recovery time [median (IQR)], days	53 (93)	61 (108.8)	48 (62)	0.552
Still ongoing, n (%)	105 (54.7%)	49 (45.8%)	56 (65.9%)	**0.005**
Loss of taste, n (%)	164 (30.2%)	111 (35.7%)	53 (22.8%)	**0.001**
Recovery time [median (IQR)], days	31 (44.3)	31 (57)	38 (40.8)	0.939
Still ongoing, n (%)	39 (23.8%)	25 (22.5%)	14 (26.4%)	0.584
Loss of smell, n (%)	143 (26.3%)	92 (29.6%)	51 (22%)	**0.047**
Recovery time [median (IQR)], days	31 (57.8)	31 (73.5)	31 (41)	0.407
Still ongoing, n (%)	39 (27.3%)	24 (26.1%)	15 (29.4%)	0.669
Hair loss, n (%)	114 (21%)	51 (16.4%)	63 (27.2%)	**0.002**
Recovery time [median (IQR)], days	92 (87.5)	92 (62)	92 (91.3)	0.163
Still ongoing, n (%)	9 (7.9%)	4 (7.8%)	5 (7.9%)	>0.999
Memory lapses, n (%)	113 (20.8%)	51 (16.4%)	62 (26.7%)	**0.003**
Recovery time [median (IQR)], days	184 (190)	253 (160)	122.5 (146.5)	0.133
Still ongoing, n (%)	94 (83.2%)	42 (82.4%)	52 (83.9%)	0.830
Sleep difficulties, n (%)	105 (19.3%)	52 (16.7%)	53 (22.8%)	0.074
Recovery time [median (IQR)], days	61 (61)	62 (61)	61 (76.5)	0.555
Still ongoing, n (%)	60 (57.1%)	28 (53.8%)	32 (60.4%)	0.499
Muscular weakness, n (%)	101 (18.6%)	53 (17%)	48 (20.7%)	0.280
Recovery time [median (IQR)], days	30 (47)	18.5 (51)	39 (71)	**0.018**
Still ongoing, n (%)	40 (39.6%)	19 (35.8%)	21 (43.8%)	0.417
Headache, n (%)	97 (17.9%)	62 (19.9%)	35 (15.1%)	0.144
Recovery time [median (IQR)], days	14 (53)	14 (28)	17 (50.5)	0.369
Still ongoing, n (%)	49 (50.5%)	25 (40.3%)	24 (68.6%)	**0.008**
Myalgia, n (%)	87 (16%)	41 (13.2%)	46 (19.8%)	**0.037**
Recovery time [median (IQR)], days	77 (91)	46 (91)	92 (59)	0.465
Still ongoing, n (%)	62 (70.5%)	26 (61.9%)	36 (78.3%)	0.093
Low-grade fever, n (%)	75 (13.8%)	50 (16.1%)	25 (10.8%)	0.077
Recovery time [median (IQR)], days	7 (8)	7 (8.5)	8 (7)	0.243
Still ongoing, n (%)	0	0	0	-
Mood changes, n (%)	68 (12.5%)	34 (10.9%)	34 (14.7%)	0.195
Recovery time [median (IQR)], days	152.5 (257.5)	122 (270)	203 (253)	0.605
Still ongoing, n (%)	49 (72.1%)	24 (70.6%)	25 (73.5%)	0.787
Gastrointestinal symptoms, n (%)	63 (11.6%)	42 (13.5%)	21 (9.1%)	0.109
Recovery time [median (IQR)], days	7 (13)	6 (10.7)	10 (17)	0.145
Still ongoing, n (%)	6 (9.5%)	5 (11.9%)	1 (4.8%)	0.654
Chest pain, n (%)	47 (8.7%)	32 (10.3%)	15 (6.5%)	0.117
Recovery time [median (IQR)], days	24 (105)	13.5 (55.8)	61 (91)	**0.035**
Still ongoing, n (%)	23 (48.9%)	15 (46.9%)	8 (53.3%)	0.680
Skin rash, n (%)	47 (8.7%)	18 (5.8%)	29 (12.5%)	**0.006**
Recovery time [median (IQR)], days	30 (43.8)	31 (71.5)	30 (15)	0.743
Still ongoing, n (%)	14 (29.8%)	4 (22.2%)	10 (34.5%)	0.372
Palpitations, n (%)	46 (8.5%)	23 (7.4%)	23 (9.9%)	0.297
Recovery time [median (IQR)], days	84 (113.8)	80 (133.5)	84 (95.5)	0.699
Still ongoing, n (%)	25 (54.3%)	11 (47.8%)	14 (60.9%)	0.375
Concentration difficulties, n (%)	40 (6.8%)	21 (6.8%)	19 (8.2%)	0.526
Recovery time [median (IQR)], days	193 (198)	163 (245.3)	193 (122)	<0.999
Still ongoing, n (%)	29 (72.5%)	14 (66.7%)	15 (78.9%)	0.385
Sore throat, n (%)	31 (5.7%)	24 (7.7%)	7 (3%)	**0.020**
Recovery time [median (IQR)], days	14 (23)	14 (19)	64.5 (7)	0.594
Still ongoing, n (%)	12 (38.7%)	7 (29.2%)	5 (71.4%)	**0.043**
At least 1 persistent symptom, n (%)	459 (84.5%)	250 (80.4%)	209 (90.1%)	**0.002**
At least 1 ongoing symptom, n (%)	309 (56.9%)	154 (49.5%)	155 (66.8%)	**<0.001**
